# Chest Fat Quantification via CT Based on Standardized Anatomy Space in Adult Lung Transplant Candidates

**DOI:** 10.1371/journal.pone.0168932

**Published:** 2017-01-03

**Authors:** Yubing Tong, Jayaram K. Udupa, Drew A. Torigian, Dewey Odhner, Caiyun Wu, Gargi Pednekar, Scott Palmer, Anna Rozenshtein, Melissa A. Shirk, John D. Newell, Mary Porteous, Joshua M. Diamond, Jason D. Christie, David J. Lederer

**Affiliations:** 1 Medical Image Processing Group, Department of Radiology, University of Pennsylvania, Philadelphia, Pennsylvania, United States of America; 2 Department of Medicine, Duke University, Durham, North Carolina, United States of America; 3 Department of Radiology, Columbia University, New York City, New York, United States of America; 4 Department of Radiology, University of Iowa, Iowa City, Iowa, United States of America; 5 Division of Pulmonary and Critical Care Medicine, Hospital of the University of Pennsylvania & Center for Clinical Epidemiology and Biostatistics, University of Pennsylvania School of Medicine, Philadelphia, Pennsylvania, United States of America; 6 Division of Pulmonary, Allergy, and Critical Care Medicine, Columbia University Medical Center, New York City, New York, United States of America; University of Chicago, UNITED STATES

## Abstract

**Purpose:**

Overweight and underweight conditions are considered relative contraindications to lung transplantation due to their association with excess mortality. Yet, recent work suggests that body mass index (BMI) does not accurately reflect adipose tissue mass in adults with advanced lung diseases. Alternative and more accurate measures of adiposity are needed. Chest fat estimation by routine computed tomography (CT) imaging may therefore be important for identifying high-risk lung transplant candidates. In this paper, an approach to chest fat quantification and quality assessment based on a recently formulated concept of standardized anatomic space (SAS) is presented. The goal of the paper is to seek answers to several key questions related to chest fat quantity and quality assessment based on a single slice CT (whether in the chest, abdomen, or thigh) versus a volumetric CT, which have not been addressed in the literature.

**Methods:**

Unenhanced chest CT image data sets from 40 adult lung transplant candidates (age 58 ± 12 yrs and BMI 26.4 ± 4.3 kg/m^2^), 16 with chronic obstructive pulmonary disease (COPD), 16 with idiopathic pulmonary fibrosis (IPF), and the remainder with other conditions were analyzed together with a single slice acquired for each patient at the L5 vertebral level and mid-thigh level. The thoracic body region and the interface between subcutaneous adipose tissue (SAT) and visceral adipose tissue (VAT) in the chest were consistently defined in all patients and delineated using Live Wire tools. The SAT and VAT components of chest were then segmented guided by this interface. The SAS approach was used to identify the corresponding anatomic slices in each chest CT study, and SAT and VAT areas in each slice as well as their whole volumes were quantified. Similarly, the SAT and VAT components were segmented in the abdomen and thigh slices. Key parameters of the attenuation (Hounsfield unit (HU) distributions) were determined from each chest slice and from the whole chest volume separately for SAT and VAT components. The same parameters were also computed from the single abdominal and thigh slices. The ability of the slice at each anatomic location in the chest (and abdomen and thigh) to act as a marker of the measures derived from the whole chest volume was assessed via Pearson correlation coefficient (PCC) analysis.

**Results:**

The SAS approach correctly identified slice locations in different subjects in terms of vertebral levels. PCC between chest fat volume and chest slice fat area was maximal at the T8 level for SAT (0.97) and at the T7 level for VAT (0.86), and was modest between chest fat volume and abdominal slice fat area for SAT and VAT (0.73 and 0.75, respectively). However, correlation was weak for chest fat volume and thigh slice fat area for SAT and VAT (0.52 and 0.37, respectively), and for chest fat volume for SAT and VAT and BMI (0.65 and 0.28, respectively). These same single slice locations with maximal PCC were found for SAT and VAT within both COPD and IPF groups. Most of the attenuation properties derived from the whole chest volume and single best chest slice for VAT (but not for SAT) were significantly different between COPD and IPF groups.

**Conclusions:**

This study demonstrates a new way of optimally selecting slices whose measurements may be used as markers of similar measurements made on the whole chest volume. The results suggest that one or two slices imaged at T7 and T8 vertebral levels may be enough to estimate reliably the total SAT and VAT components of chest fat and the quality of chest fat as determined by attenuation distributions in the entire chest volume.

## Introduction

Mortality following lung transplantation is high; nearly 50% die within 5 years after transplantation [1]. Given the scarcity of organs and the high long-term mortality, careful selection of recipients is imperative. The lung transplant community therefore follows carefully-chosen selection guidelines to exclude those at high risk of early post-operative death [2]. Presently, obesity, defined as a body mass index (BMI) greater than 30 kg/m^2^, is considered a relative contraindication to lung transplantation due to its associations with early mortality [2–4] and primary graft dysfunction [4], although recent evidence suggests that BMI is a poor measure of adiposity in patients with advanced lung disease [5]. Indeed, even in healthy adults, BMI fails to identify many patients with obesity [6]. Furthermore, BMI cannot describe fat distribution in different body regions. Previous research has shown that BMI alone cannot differentiate between obese phenotypes even though body composition (differences in fat distribution given the same BMI) may indicate different phenotypes of obese subjects [7–9].

The metabolic and inflammatory effects of adipose tissue vary by adipose tissue depot. Abdominal visceral adipose tissue (VAT) mass (i.e., omental and mesenteric fat) has been associated with the metabolic syndrome (dyslipidemia, hypertension, and insulin resistance) and atherosclerosis more strongly than subcutaneous adipose tissue (SAT) mass [10–18]. Therefore, abdominal VAT is considered "metabolically active" and more relevant to human disease than SAT. There has been little previous investigation of thoracic VAT (i.e., mediastinal and pericardial fat). In the Framingham Heart Study, thoracic VAT has been linked to aortic atherosclerotic disease (independent of abdominal VAT) [15], and cardiometabolic risk and disease [19, 20]. Epicardial adipose tissue has been associated with atherosclerosis and insulin resistance [21–24].

We designed the Lung Transplant Body Composition study to examine associations between specific measures of body composition—such as adipose tissue mass and distribution—with primary graft dysfunction (PGD) and other outcomes before and after lung transplantation. The roles of thoracic and abdominal VAT in the development of PGD have not been studied. Thoracic VAT may be particularly important for the lung, since venous blood draining thoracic VAT is sent directly to the pulmonary circulation via the right ventricle. In our study, we have chosen to measure thoracic VAT using full thoracic CT imaging, which is routinely performed in the clinical setting for lung transplant candidates, as well as abdominal VAT using a single 1cm (2 x 5mm collimation) axial CT scan at L4/L5.

In the quantification of body SAT and VAT components via CT or MRI, two fundamental questions arise: Should quantification be performed on a single slice (two-dimensional, 2D) or on the whole 3D body region? How should the SAT and VAT components be segmented on the images for the 2D and 3D approaches? This paper focuses on the first issue. We emphasize that automated fat delineation, whether whole fat or as SAT and VAT components, is quite challenging in any body region, especially in the thorax, on both CT and MRI images.

The first issue is motivated by pragmatic considerations in the standard clinical setting since single slice imaging can reduce patient discomfort, cost, imaging time (in case of MRI), and radiation (in case of CT). It, however, raises the following fundamental questions for quantification of fat in the thorax. (1) What is the proper anatomic definition of SAT and VAT in the thorax? (2) How good is the 2D area estimate as a marker of 3D volume in the thorax? (3) At what anatomic slice location in the chest this approximation is the best? (4) Do SAT and VAT components behave differently in terms of the best slice location? (5) Do their attenuation distributions differ in different disease conditions and between 2D and 3D approaches? (6) How do chest 2D and 3D quantifications compare to 2D quantification in the abdomen and thigh? (7) What is their relationship to BMI? The main contribution of this paper is to seek answers to these questions, which have not been addressed in the literature and are important in the context of lung transplant surgery. Since pre-surgery CT scans are obtained for all patients, these questions can be answered using information contained within already existing data sets. Moreover, measurements made directly from CT scans not only provide accurate fat assessment but also obviate the need for extra scans such as DXA, potentially leading to lower cost, decreased patient radiation exposure, and decreased time and inconvenience to patients.

The proposed approach employs a previously developed method called *Standardized Anatomic Space* (SAS) [25] for consistently mapping axial CT slices from different subjects to the same homologous anatomic location. It was demonstrated in [25] that such a mapping becomes essential to meaningfully analyze 2D-slice-area to 3D-volume correlations and HU distributions for determining the best slice locations.

## Materials and Methods

### Image data sets

This image analysis study was conducted following approval from the Institutional Review Board at the University of Pennsylvania. Written consent was obtained from every participating patient. The consent form and the consent procedure were approved by the IRBs at participating institutions. (The annual IRB approvals at Penn, Duke, and Columbia are valid until 6/12/2017, 6/27/2017, and 10/25/2017, respectively.) Unenhanced CT image data sets from 40 adult lung transplant candidates, including 16 patients with idiopathic pulmonary fibrosis (IPF) and 16 patients with chronic obstructive pulmonary disease (COPD) were analyzed. Every subject had an unenhanced chest CT scan as well as research single slice CT scans of the thigh at mid-level and a single slice CT scan of the abdomen at the L5 level as part of an NHLBI-funded and IRB-approved prospective study at 3 lung transplant centers: Columbia, Penn, and Duke. Table 1 lists key patient demographic information by gender and under three disease groups—COPD, IPF and Other. Certain parameters related to Pulmonary Function Tests are also listed in the table.

**Table 1:** Patient demographics. Mean (median) and standard deviation values are listed.

Group	n	Age	BMI (Kg/m^2^)	FVC (L)	FEV1 (L)	RV (L)	TLC (L)
**COPD**	16	58.56	26.49	2.74	1.33	3.49	6.48
	12 M	(61.00)	(26.99)	(2.86)	(1.08)	(3.42)	(6.18)
	4 F	8.94	4.87	0.90	0.76	1.90	1.86
**IPF**	16	62.38	27.09	2.17	1.55	2.08	4.50
	14 M	(65.50)	(27.46)	(2.11)	(1.60)	(1.38)	(4.25)
	2 F	9.95	3.75	0.59	0.63	1.57	1.59
**Other**	8	48.50	24.41	2.79	1.96	2.00	4.88
	7 M	(55.50)	(23.20)	(1.90)	(1.19)	(1.20)	(4.23)
	1 F	15.01	4.70	1.47	1.20	1.52	2.24

Chest scans were performed during full inspiration with multi-detector row CT scanners (Siemens Sensation, Definition, FLASH, and AS, Siemens Medical Systems; GE VCT & HD, General Electric) with 64 to 128 detector rows, 16 to 128 x 0.6 to 1.25 mm slice collimation, kVp of 120, approximately 110–120 effective mAs for Siemens machines and 80–500 mA for GE machines with CareDose on, gantry rotation time of 0.4–0.5s, reconstructed with I50f, B30, or B35 kernel for Siemens machines and Standard kernel for GE machines. The image size was 512×512 with 50–70 slices and voxel size that varied from 0.70×0.7×5 mm^3^ to 0.97×0.97×5 mm^3^. Abdomen and thigh scans were performed with multi-detector row CT scanners (Siemens Sensation, Definition, FLASH, and AS, Siemens Medical Systems; GE VCT & HD, General Electric) with 64 to 128 detector rows, 2 x 5 mm slice collimation, kVp of 120, 200 effective mAs for Siemens machines and 300 mA for GE machines, gantry rotation time of 1.0 s without dose modulation, reconstructed with B35 kernel for Siemens machines and Standard kernel for GE machines. Standard QA procedures were followed in all institutions to ensure quality and standard of the clinical scans.

Our approach to answering the above questions consists of the following steps: (1) SAT and VAT definition and delineation on CT images; (2) mapping slices to anatomically homologous locations through the SAS approach; (3) fat quality analysis through attenuation distributions; (4) fat area-to-volume and other correlative analyses. These steps are described below.

#### (1) SAT and VAT definition and delineation on CT images

**Fig 1:**
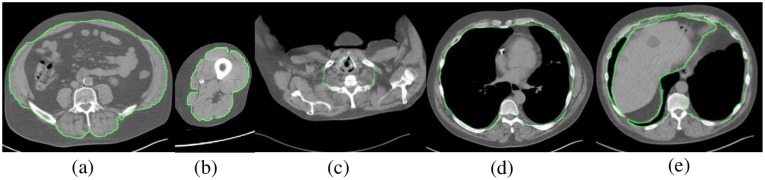
Definition of SAT-VAT interface illustrated through boundary contour drawn on slices: (a) at L4-L5 level in lower abdominal region, (b) through mid-thigh level, (c) in upper thorax, (d) in mid thorax, and (e) in lower thorax at level of diaphragm.

For all subjects, the thoracic body region was defined consistently as extending from 15 mm superior to the apex of the lungs to 5 mm inferior to the base of the lungs. All patient CT images were accordingly trimmed to include just this standardized body region. The interface between SAT and VAT in the abdomen and thigh is much easier to define anatomically, as illustrated in Fig 1(a) and 1(b), than in the chest [26, 27]. In the superior portion of the thorax, no clear demarcation exists. In the inferior portion of the thorax, where axial slices pass through the curved diaphragm, abdominal visceral fat appears in the slices which must be excluded. Generally, we define the thoracic SAT-VAT interface as the interior surface of the rib cage; fat within this surface is defined to be VAT and that external to this surface is defined as SAT for all slices which are superior to the diaphragm. For slices passing through the diaphragm, the definition of SAT remains the same. The VAT component, however, is modified in these slices by removing the visceral fat located within the abdomen. These definitions are illustrated in Fig 1(c), 1(d) and 1(e) for a slice in each of the superior, mid, and inferior portions of the thorax.

The interface between SAT and VAT in the thorax was drawn by using the Live Wire contouring tool of the CAVASS software system [28] following the above definition. The VAT component internal to this interface was segmented by setting a HU threshold interval of [-170, -40] followed by a morphological opening operation to remove isolated pixels. To segment the SAT component, the entire thoracic body region was first segmented by a threshold operation followed by filling air cavities automatically via connected component labeling. The entire adipose tissue mass within the body region so delineated was then segmented by using the above threshold interval. The computed VAT component was then removed from this mass to obtain the SAT component. In some cases (10 out of 40 among our data sets), a further step of manual correction was needed for removing additional pseudo fat voxels appearing within medullary bone. The approach is the same for all three body regions.

To assess the reproducibility of fat volume and area measurements, two operators who have been trained to recognize the SAT/VAT interface as defined above performed segmentation on 5 data sets twice for each body region. The intra- and inter-operator variability (precision) in volume and area measurements are assessed from these delineations following the framework described in [29], where precision is defined as |S1∩S2| / |S1∪S2|, where S1 and S2 are binary segmentations in two repeated trials, and |.| denotes volume (or area).

From the segmented volume image/slice image, volume and area of the SAT and VAT components were computed directly. We normalized fat measurements by using the diagonal of a box that encloses the thoracic skeleton. If L denotes this normalizing length for a subject, then fat volumes were normalized by dividing by L^3^, and fat areas were normalized by dividing by L^2^. For any given chest slice, the SAT/VAT area in that slice was also computed from the segmentation results in addition to the volume of those components in the entire chest. These areas are needed to determine the slice for which the SAT/VAT components of area show the best correlation with the respective volumes.

#### (2) Mapping slices to anatomically homologous locations through the SAS approach

**Fig 2:**
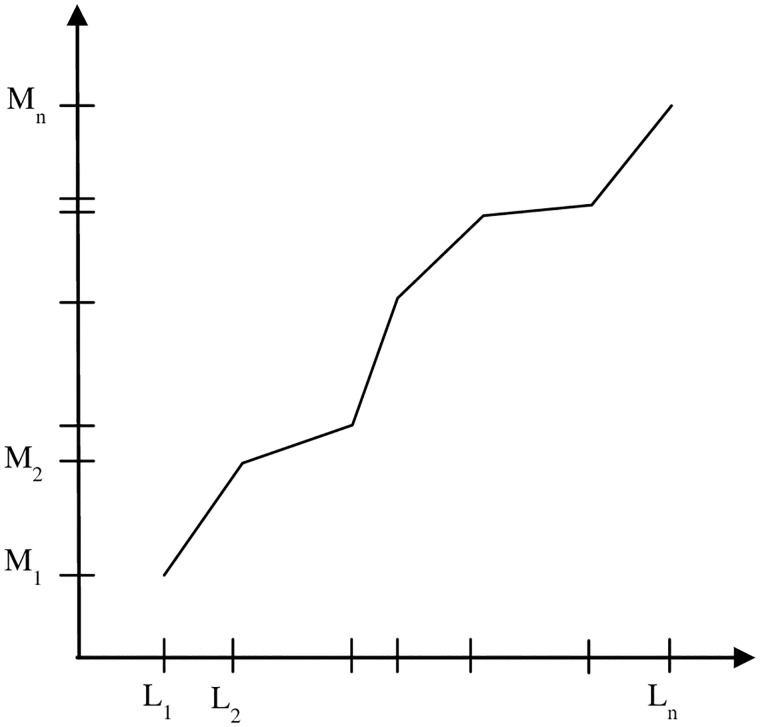
Non-linear mapping of slice locations to standardized anatomic space.

The SAS approach [25] is landmark based. It uses mid-axial levels of the vertebral bodies as landmarks in the cranio-caudal direction. The landmarks are selected on 3D renditions of the skeletal structure. The approach consists of two stages—*calibration* and *transformation*. The purpose of calibration is to estimate mean locations of the landmarks, denoted M_1_, …, M_n_, by using a few reference patient data sets. This stage is executed only once and not performed while analyzing each patient data set. In the transformation stage, the same landmarks are identified on each patient image. These landmarks are denoted L_1_, …, L_n_. A non-linear (piece-wise linear) mapping is defined between the patient landmarks and the estimated mean locations as illustrated in Fig 2. This mapping defines the anatomic location of any given slice in the image data set of any patient in the standardized space. The mapping allows selecting the same anatomically homologous slices in different patient studies. The acquired slice images are not modified in any manner in this mapping process (such as interpolation, etc.). Only the slice number within the acquired stack of slices is determined for each subject for each anatomic location in the standardized space. We selected 9 landmarks—through the middle of T2, T3, …, T10 and used 20 patient image sets for the calibration step.

#### (3) Fat quality analysis through attenuation distributions

To study the tissue properties of fat, several key parameters derived from attenuation histograms within SAT and VAT components in the chest, abdomen, and thigh were analyzed. The parameters studied were: mean (H_m_), median (H_md_), mode (H_p_), and the lower and upper quartile (H_*l*q_ and H_uq_, respectively) of the attenuation values (all expressed in HU).

#### (4) Correlative analyses

We present SAT and VAT quantities as normalized values, and attenuation histogram properties are listed in HU only for the VAT region. Pearson correlation coefficient (abbreviated PCC) was analyzed between several pairs of parameters treated as random variables. Correlation coefficient from PCC between two random variables A and B will be denoted by ρ(A, B). A and B were selected from among fat slice-area, fat volume, BMI, and attenuation distribution parameters as listed in Table 2. As an example, ρ(VV_C_, VA_A_) denotes PCC between VAT volume in chest and VAT area in abdominal slice.

**Table 2:** Definition of variables employed in correlative analysis.

Variable		Description
BMI		Body Mass Index = weight in kg / (height in meters)^2^
Normalized area	SA_A_	SAT area in abdominal slice
	SA_T_	SAT area in thigh slice
	SA_C_	SAT area in a slice in the chest
	VA_A_	VAT area in abdominal slice
	VA_T_	VAT area in thigh slice
	VA_C_	VAT area in a slice in the chest
Normalized volume	SV_C_	SAT volume in chest
	VV_C_	VAT volume in chest
Attenuation histogram parameters(in HU)	SVH_C,m_,	SAT histogram parameters in chest volume: mean,
	SVH_C,md_, SVH_C,p_,	median, mode
	SVH_C,*l*q_, SVH_C,uq_	lower and upper quartile
	SAH_C,m_,	SAT histogram parameters in chest slice: mean,
	SAH_C,md_, SAH_C,p_	median, mode
	SAH_C,*l*q_, SAH_C,uq_	lower and upper quartile
	VVH_C,m_,	VAT histogram parameters in chest volume: mean,
	VVH_C,md_, VVH_C,p_,	median, mode
	VVH_C,*l*q_, VVH_C,uq_	lower and upper quartile
	VAH_C,m_,	VAT histogram parameters in chest slice: mean,
	VAH_C,md_, VAH_C,p_	median, mode
	VAH_C,*l*q_, VAH_C,uq_	lower and upper quartile

The location of a single thoracic slice in the standardized space where volume-to-area PCC values ρ(SV_C_, SA_C_) and ρ(VV_C_, VA_C_) reach maximum were determined separately for the SAT and VAT components. We will refer to these slices as “best slices” for chest. In a similar manner, the “best 2-slices” and “best 3-slices” were determined by finding the locations of two and three slices whose area sum correlated maximally with the volume. Note that in these cases, the slices may not be contiguous in space.

Among the 38 variables in Table 2, although many pairings are possible for correlative analysis, we considered only the following pairs which made intuitive sense: chest volume of SAT and VAT with BMI and SAT and VAT area in the abdomen and thigh; chest best slice area for SAT and VAT with BMI and SAT and VAT area in the abdomen and thigh; attenuation histogram parameters of chest SAT and VAT 3D region with attenuation histogram parameters of chest best slice for SAT and VAT.

We used unpaired t-tests to compare variables COPD and IPF groups. We also present P values from Mann-Whitney U tests in parentheses.

## Results

**Fig 3:**
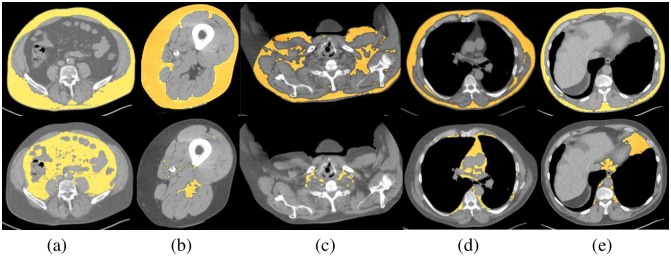
SAT (top row) and VAT (bottom row) segmentation from images of one subject overlaid on slice display for: (a) abdomen, (b) thigh, (c) slice in upper thorax, (d) slice in mid thorax, and (e) slice in lower thorax at level of diaphragm.

Fig 3 shows the segmentations obtained on the chest, abdominal, and thigh images of one subject where the segmentations are overlaid on the slice display. For chest, three slices are displayed (along the lines of Fig 1), one each from the superior, mid, and inferior aspect of the thorax to illustrate the varying distribution of fat. The intra- and inter observer precision in fat measurement found in our repeatability experiments are listed in Table 3.

**Table 3:** Intra- and inter-operator repeatability (precision [44]) in SAT and VAT measurement in repeated segmentations.

		Chest (volume)	Abdomen (area)	Thigh (area)
**SAT**	**Intra**	0.983	0.992	0.975
	**Inter**	0.945	0.978	0.945
**VAT**	**Intra**	0.975	0.998	0.988
	**Inter**	0.965	0.991	0.965

**Fig 4:**
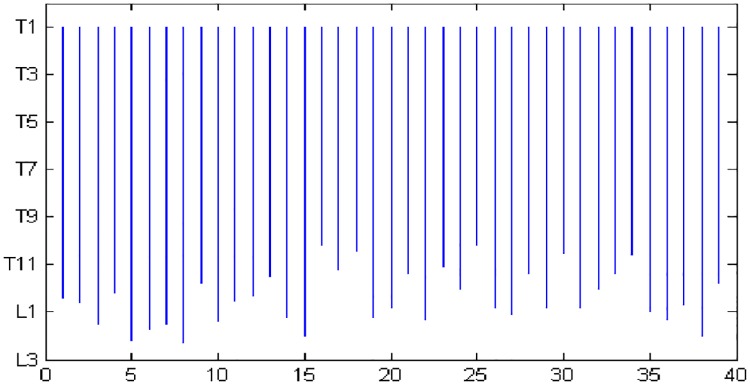
Anatomic locations of chest slices for 40 subjects. Abscissa shows subject number and ordinate indicates the extent of the thorax in cranio-caudal direction for different subjects.

To illustrate the anatomic variability that exists among subjects, in Fig 4 we plot schematically the locations of the midlevel of vertebral bodies in the cranio-caudal (vertical) direction for all subjects considered in the study. The top and the bottom of the vertical line for each subject indicate the extent of the thoracic body region in relation to the vertebral bodies. In all subjects, the thoracic region as defined in this paper starts from roughly the T1 vertebra. However, the locations of the inferior boundary show significant variability. For some subjects (5, 8, 15, 38), the thorax extends up to or beyond L2, while for others (16, 18, 25, 30) it barely reaches T10. Some of the lower slice locations approaching L2 were from COPD patient data sets (22, 27, 29, 35, 36, 38) and others were from the IPF group (5, 8, 15). Thus, seemingly, the location variability is not specific to one patient group. The distribution of the number of subjects whose inferior thoracic boundary falls at different vertebral levels is as follows. T9-T10: 1; T10-T11: 6; T11-T12: 9; T12-L1: 11; L1-L2: 9; L2-L3: 4.

**Fig 5:**
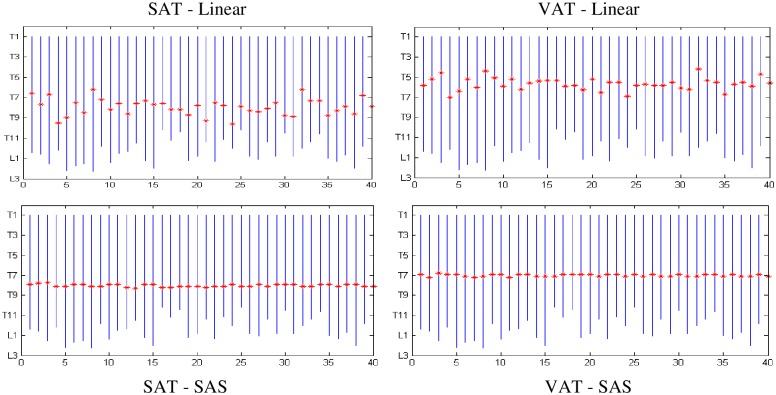
Best slice location found by linear mapping (top row) and SAS mapping (bottom row) for SAT (left) and VAT (right) shown by "*" for each subject.

It was shown in [25] that linear mapping will not properly handle the non-linearity that exists in slice locations. This variability seems to be even higher in the abdominal region (cf. Fig 4 in [25]). Fig 5 further illustrates this point by plotting by “*” the locations of the best single slice found by the SAS method for different subjects in comparison to linear mapping where slice locations are interpreted in a proportionate manner based on the first and last slices of the defined thoracic volume image. The best locations found by linear mapping vary from subject to subject for both SAT and VAT anywhere from T6 to T10 for SAT and T4 to T7 for VAT, while the location is constant for the SAS method—T8 for SAT and T7 for VAT.

The various correlations studied are summarized in Table 4. Volume-to-area correlation in the chest has three entries corresponding to the number of best slices considered (1, 2, or 3) for both SAT and VAT. All entries in the table when referring to a single slice (or double or triple slices) in the chest represent optimum correlation corresponding to the best slice(s). For example, the last entry in the table ρ(BMI, VA_C_) means PCC between BMI and the VAT area in the best chest slice which recorded maximum VAT volume-to-area correlation. To check if the correlations would be different if we used Spearman rank correlation coefficient (abbreviated SCC) instead of PCC, on a subset of the set of all pairs of variables we carried out SCC analysis. These additional SCC values are shown in parenthesis in Table 4.

**Table 4:** Summary of correlations from PCC. Attenuation histogram property analysis and BMI-to-(SAT/VAT-volume/area) correlative analysis with PCC (and SCC in parenthesis).

Group	ρ(.)		Value	Group	ρ(.)	Value
Volume-to-area	SV_C_, SA_A_		0.73	(Attenuation histogram property-volume)-to-(attenuation histogram property-slice) (continued)	SVH_C,p_, SAH_C,p_	0.90 (0.86)
	SV_C_, SA_T_		0.52		SVH_C,*l*q_, SAH_C,*l*q_	0.97 (0.96)
	SV_C_, SA_C_	1 sl	0.97		SVH_C,uq_, SAH_C,uq_	0.98 (0.94)
		2 sl	0.97		VVH_C,m_, VAH_C,m_	0.77 (0.78)
		3 sl	0.98		VVH_C,md_, VAH_C,md_	0.79 (0.81)
	VV_C_, VA_A_		0.75		VVH_C,p_, VAH_C,p_	0.42 (0.41)
	VV_C_, VA_T_		0.37		VVH_C,*l*q_, VAH_C,*l*q_	0.85 (0.85)
	VV_C_, VA_C_	1 sl	0.86		VVH_C,uq_, VAH_C,uq_	0.77 (0.75)
		2 sl	0.92	BMI-to-(SAT/VAT-volume/area)	BMI, SV_C_	0.65 (0.59)
		3 sl	0.95		BMI, VV_C_	0.28 (0.26)
Area-to-area	SA_C_, SA_A_		0.74		BMI, SA_A_	0.59 (0.64)
	SA_C_, SA_T_		0.59		BMI, SA_T_	0.41 (0.51)
	VA_C_, VA_A_		0.41		BMI, VA_A_	0.3 (0.25)
	VA_C_, VA_T_		0.4		BMI, VA_T_	0.37 (0.33)
(Attenuation histogram property-volume)-to-(attenuation histogram property-slice)	SVH_C,m_, SAH_C,m_		0.98 (0.87)		BMI, SA_C_	0.63 (0.56)
	SVH_C,md_, SAH_C,md_		0.98 (0.96)		BMI, VA_C_	0.36 (0.34)

The locations of the best slice(s) found for SAT and VAT in the chest are listed in Table 5. T7 means the location is at the middle of the T7 vertebra and T6-T7 means the location is between T6 and T7 vertebra. To check if the best single slice location differed for the two patient subgroups COPD and IPF, we carried out the entire SAS analysis separately on these two patient groups. For SAT, the best slice locations found were: T8-T9 (**ρ** = 0.99) and T8 (**ρ** = 0.98) for IPF and COPD, respectively. For VAT, the locations were: T7-T8 (**ρ** = 0.85) and T8 (**ρ** = 0.87) for IPF and COPD, respectively. Thus, the best slice locations were almost the same for the two patient groups despite the large variation in slice locations in the original data sets for the two groups.

**Table 5:** Locations of chest slices with maximum volume-to-area correlation.

Number of slices considered	Locations for SAT	Locations for VAT
1	T8	T7
2	T6-T7, T9-T10	T2, T5-T6
3	T2, T8, T9-T10	T2, T5-T6, T8

Although the number of female patients is small in our study, we performed an analysis to determine if there was any gender-specific difference in chest SAT and VAT volumes and the best slice locations. The [mean, median, SD] values of normalized SAT and VAT volumes for the female and male groups were as follows. SAT: [0.26, 0.28, 0.13] and [0.14, 0.13, 0.06]; VAT: [0.02, 0.02, 0.01] and [0.02, 0.02, 0.01]. The difference in VAT between the two genders is not significant, although the difference in SAT was significant (p < 0.05). The best slice location found for SAT is T8 for the female group and the mid-level between T8 and T9 for the male group, while for VAT the location is the same (T7) for the two groups.

In Table 6, we summarize the computed fat volume/area and the attenuation histogram properties for the COPD and IPF groups derived from the whole volume as well as from the best slice. The BMI values for the two groups were 26.49±4.87 and 27.09±3.75 kg/m^2^, respectively, showing no statistically significant difference. The attenuation histogram properties in the SAT region did not show statistical differences between the two groups, and are not shown in the table. However, the median attenuation of VAT in patients with COPD was statistically significantly lower compared to that of patients with IPF. Results from Mann-Whitney U test show similar trends. Although this difference in attenuation distribution constitutes a “side analysis” in this paper, it signals the need for future detailed analyses of tissue characteristics in different disease conditions and what they may portend for lung transplantation.

**Table 6:** Fat volume, area, and attenuation histogram properties on chest CT for COPD (n = 16) and IPF (n = 16) groups.

Variable	From 3D volume			From best slice		
	COPD	IPF	P value	COPD	IPF	P value
SAT	0.16 ± 0.11	0.16 ± 0.07	0.97 (0.64)	0.17 ± 0.14	0.20 ± 0.09	0.60 (0.32)
VAT	0.02 ± 0.01	0.02 ± 0.01	0.46 (0.44)	0.03 ± 0.02	0.03 ± 0.02	0.32 (0.42)
H_m_	-73 ± 6	-66 ± 5	0.00 (0.00)	-72 ± 6	-67 ± 5	0.01 (0.01)
H_md_	-73 ± 8	-65 ± 6	0.01 (0.01)	-71± 8	-65 ± 6	0.01 (0.01)
H_p_	-76 ± 13	-69 ± 11	0.09 (0.08)	-73 ± 19	-59 ± 19	0.04 (0.04)
H_*l*q_	-96 ± 10	-87 ± 10	0.01 (0.01)	-96 ± 10	-88 ± 9	0.02 (0.02)
H_uq_	-47 ± 7	-42 ± 5	0.03 (0.04)	-46 ± 8	-43 ± 7	0.17 (0.22)

A similar analysis carried out on abdominal and thigh SAT and VAT areas and VAT attenuation properties showed no significant difference between the IPF and COPD groups. However, the difference in mean and low quartile of SAT attenuation properties were significant (p < 0.04 and p < 0.05) between the IPF and COPD groups. For the third “other” (non-IPF, non-COPD) group, the mean SAT and VAT attenuation values were as follows. Chest: -82.1 (SD 4.2), -67.5 (SD 5.9); Abdomen: -83.8 (SD 3.7), -75.1 (SD 4.8); Thigh: -87.4 (SD 4.9), -62.4 (SD 7.5). No significant difference was found in SAT and VAT areas and VAT attenuation properties between “other” and IPF or COPD groups. However, for SAT at thigh, the difference in the mean, median, mode, and high quartile of attenuation properties between the 3rd group and IPF group is significant (p < 0.04) but not for the COPD group.

## Discussion

This study attempted to answer several key questions related to the process of estimating the amount and quality of SAT and VAT components of fat via CT imaging in patients who are candidates for lung transplant surgery. It also performed a preliminary investigation of how these parameters may differ between two important patient subgroups. The process of quantification started with a consistent definition of the thoracic body region and the chest SAT/VAT interface. An interactive method to segment the two components was implemented. The concept of Standardized Anatomic Space, previously developed in the context of abdominal fat quantification [25], was applied to the thorax. Its adaptation was essential for consistently choosing the same anatomic slice in the thorax in all patients to study the relationship between properties measured from the whole volume and from a single slice.

In answering the questions raised earlier in the paper, we note from Figs 4 and 5 that there is considerable subject-to-subject variation in the anatomic location of slices in the thorax (although less than that encountered in the abdomen [25]), and the SAS method effectively overcomes this in selecting corresponding slices in different subjects automatically. The SAS approach considers all slice locations instead of selected locations, such as L4-L5 or 5 cm above L4-L5 as in [30–37], or seven selected locations tested in [38] for abdomen. The optimal location may be variable from patient to patient and SAS can find the appropriate location. We are not aware of any past efforts to find the best slice location in thoracic fat analysis. A somewhat related work was reported by Tran et al. [39] whose aim was to determine the ability of epicardial fat area measured on a single CT slice at the level of the ostium of the left main coronary artery in predicting obstructive coronary artery disease. They found that both epicardial fat area and volume were associated with the disease and fat area to volume correlation was 0.89 at this level. Their analysis did not standardize the anatomic space to account for possible anatomic variations and they did not examine the slice level that yielded the best area-to-volume correlation.

In this study, the slice location that yields maximal correlation between area and volume is found to be different for SAT and VAT—at the mid-level of T8 for SAT and at T7 for VAT. These locations are very similar for the COPD and IPF patient groups, despite the differences in these patient groups in slice location distribution. Similarly, although from a small sample, no difference in the best slice location for VAT was found between male and female subjects, and the location for SAT was also very similar for the two genders. Area from the best slice shows very high correlation with volume: 0.97–0.99 for SAT and 0.85–0.87 for VAT. Inclusion of 2 and 3 best slices does not influence this correlation much for SAT, although VAT correlation improves to 0.92 and 0.95, respectively. Correlations of chest fat volume with fat area from the abdominal slice at the L5 level is modest for SAT (0.73) and VAT (0.75). However, these volume-to-area correlations are quite weak for thigh for SAT (0.52) and especially for VAT (0.37). Interestingly, the correlation of BMI with chest fat volume (Table 4) is modest for SAT (0.65) but weak for VAT (0.28), and the behavior remains the same for measures derived from the best slice. The behavior of volume to best slice relationship observed for fat attenuation parameters is similar to the above fat quantity measures.

Note that the best single slice location for SAT and VAT to express 3D abdominal fat burden is not at L5 but rather at T12-L1 for SAT and at L3-L4 for VAT [25]. It is difficult to ascertain whether our estimated chest-to-abdominal fat correlations would have improved if abdominal slices were available at those optimal locations. Unfortunately, our abdominal research scans were obtained following the clinical practice of imaging at the L5 level for an abdominal slice. Since high correlation does not imply ability of the variables to predict (whatever may be the prediction task), the more interesting question is whether measurements from the best slice from chest at the T7-T8 levels (or from the best slice in abdomen) will be able to predict outcome of surgery or donor suitability, etc., when compared to whole chest fat volume. Exploration of this avenue is one of our future goals.

Fat attenuation histogram properties listed in Table 6 reveal that there is significant difference in the distributions of attenuation values between the COPD and IPF groups for VAT, the former being shifted to lower values compared to the latter. Interestingly, the best slice carries this trend faithfully. (Note that this sort of detailed tissue analysis can be carried out only using CT (or MRI) but not DXA or BMI as measurement modalities.) Considering the observation that the behavior of chest CT volume is carried over to that in the best slice for correlation with fat area in the abdomen and thigh and BMI as well, the best slice selected via the SAS approach seems like an effective marker of the entire chest volume. This may make it possible in the future to image just a couple of slices at the T7-T8 level for determining both the quantity and quality of fat in the entire chest.

Measurement of adipose tissue mass can be accomplished using a variety of tools. For instance, dual energy x-ray absorptiometry (DXA), an x-ray projection imaging modality commonly used for assessing body composition [40], can be used to assess body fat content [41, 42]. Yet, superimposition from the third dimension in the direction of projected x-rays prevents resolution of the thickness in the third dimension in different tissue regions in this modality. Hence, DXA measurements contain contributions from other tissues, leading to errors in estimation compared to true three-dimensional (3D) distribution of fat mass. Estimation of fat mass and distribution in the true 3D space therefore typically requires cross-sectional imaging, including computed tomography (CT) and magnetic resonance imaging (MRI) [43]. This is mainly because these imaging modalities do not have the conceptual drawbacks associated with measurement via BMI and DXA since both CT and MRI resolve tissue composition at every volume element (voxel) inside the body. It has been shown that quantitatively DXA and CT measurements differ considerably and hence cannot be used interchangeably [44].

One limitation of this study is the small sample size considered. This was dictated by the manual labor involved in defining the SAT-VAT interface in the thorax, which is a challenging problem, although we are currently developing anatomy-model-based strategies with the goal of making this step more efficient and to bring it to a production-mode level. As hinted above, our future work will include investigation of image-derived parameters, preferably from a few optimally selected slices, which have the best predictive ability to prognosticate clinical parameters and outcome.

In conclusion, this paper makes two key contributions: (1) By adapting the SAS approach, previously developed for the abdomen, to the chest, we demonstrated a method of consistently and optimally selecting specific slices in the chest whose measurements may be used as markers of similar measurements made on the whole chest volume. (2) In applying this method for fat quantification, we demonstrated that one or two slices imaged at T7 and T8 vertebral levels may be enough to estimate reliably the total SAT and VAT components and the quality of fat as determined by attenuation distributions in the chest volume. These concepts and results may be useful in the future for the effective study, management, and follow up of lung transplant candidates. To the best of our knowledge, such a methodological study to characterize fat quantity and quality has not been carried out in the past, especially in the chest and as related to lung transplantation.
